# Integrating environmental variables and land use types to assess the habitat suitability and atractylodin content of two *Atractylodis Rhizoma* species under climate change

**DOI:** 10.3389/fpls.2026.1866314

**Published:** 2026-07-28

**Authors:** Xingtian You, ChaoHui Wang, Zikang Lu, Xiangrui Fu, Yao Tong, Yongxing Song, Donglai Ma, Jixia Yang

**Affiliations:** 1 Hebei University of Traditional Chinese Medicine, Shijiazhuang, Hebei, China; 2 International Joint Research Center on Resource Utilization and Quality Evaluation of Traditional Chinese Medicine of Hebei Province, Shijiazhuang, Hebei, China

**Keywords:** Atractylodis Rhizoma, Atractylodin, climate change, land use type, quality variation

## Abstract

**Introduction:**

*Atractylodis Rhizoma*, a traditional Chinese medicinal herb, has significant medicinal, ecological, and scientific value; however, global climate change is altering its geographical distribution and quality.

**Methods:**

On the basis of species occurrence data, in this study, bioclimatic, topographic, and soil variables, as well as land use types, were integrated to predict the potentially suitable habitats of *Atractylodes lancea* (AL) and *Atractylodes chinensis* (AC) in China under current and future climate scenarios, and future stable quality distribution areas were proposed.

**Results:**

The results revealed that AL was influenced primarily by the precipitation of the driest quarter (Bio17), temperature seasonality (Bio4), and mean temperature of the coldest quarter (Bio11), whereas AC showed the strongest response to the precipitation of the wettest quarter (Bio16), temperature seasonality (Bio4), and precipitation of the warmest quarter (Bio18). Under current climatic conditions, AL was primarily distributed in the southern regions of the middle and lower Yangtze River Basin, whereas AC occurred mainly in the boundary areas between semiarid and arid regions, primarily in central and northeastern China. Under future climate warming scenarios, the total suitable area of AL is projected to decline and shift southeastward, whereas that of AC is projected to contract and migrate northeastward. The atractylodin content in AL was primarily associated with variables such as isothermality (Bio3) and temperature seasonality (Bio4), whereas atractylodin accumulation in AC was influenced mainly by the precipitation of the driest quarter (Bio17) and temperature seasonality (Bio4). The stable quality distribution areas of AL were primarily located in Central China, whereas those of AC were mainly distributed in North China. Under all the climate scenarios and across all the time periods considered, dryland and forestland were consistently identified as the most suitable land use types for both *Atractylodis Rhizoma* species.

**Discussion:**

These findings may provide a scientific basis for the conservation, rational introduction, and high-quality cultivation of AL and AC, as well as for future strategies to mitigate the potential impacts of climate change on these medicinal species.

## 1 Introduction

Climate change plays a crucial role in plant growth and development (O’Connor et al., 2020). Research has indicated (Bi et al., 2025) that the global average temperature has risen significantly compared with that of the previous century and that ongoing warming is altering the geographic ranges of species, resulting in a marked contraction or complete disappearance of suitable habitats for medicinal plants. The growth of medicinal plants is closely linked to local environmental conditions, which in turn influence changes in their chemical composition (Alum EU, 2024). Therefore, quantifying the impact of climate change on the distribution and quality of medicinal plants and predicting their potentially suitable habitats and high-quality cultivation zones are crucial (Takubessi et al., 2025). Such projections can guide the standardized cultivation of geoauthentic medicinal species and help stabilize the market supply.

*Atractylodis Rhizoma*, the dried rhizome of *Atractylodes lancea* (Thunb.) DC. (AL) or *Atractylodes chinensis* (DC.) Koidz. (AC), belongs to the Asteraceae family and has the effects of eliminating dampness, invigorating the spleen, expelling wind and dispelling cold, and improving eyesight (Commission CP, 2020). Recent research has indicated that the pharmacological effects of *Atractylodis Rhizoma* include not only effects against gastric ulcers, intestinal diseases, and acute lung injury but also the treatment of arthritis and other symptoms (Guo et al., 2024). The chemical constituents of *Atractylodis Rhizoma* are highly diverse and are primarily composed of volatile oils and secondary metabolites, especially sesquiterpenoids (atractylone, hinesol, and β-eudesmol) and polyacetylenes (atractylodin) (Zhang et al., 2021). Among these constituents, atractylodin is recognized as a representative polyacetylene compound and is officially designated as a quality marker in the Chinese Pharmacopoeia. As a key bioactive component, atractylodin has been reported to exhibit multiple pharmacological activities, including anti-inflammatory (Heo et al., 2023), antioxidation (Chen et al., 2021a), immune regulatory (Lin et al., 2020), and anticancer effects (Zhong and Zhang, 2024). Importantly, because of its established role in quality control standardization and its reliable quantifiability, atractylodin was selected as the target compound for quality evaluation in this study. Owing to the depletion of wild *Atractylodis Rhizoma* resources, efforts to meet market demand have led to the introduction and cultivation of this species in new regions, resulting in substantial changes in its morphological traits and chemical composition; thus, it can be distinguished into two species: AL and AC (Ke et al., 2025). AL predominantly occurs in warm and humid regions, whereas AC is primarily found in colder and drier environments, indicating substantial environmental differences between the two species (Zhang et al., 2023). Such environmental heterogeneity is expected to influence plant growth conditions and may also affect the accumulation of key secondary metabolites, including atractylodin, thereby linking environmental conditions with the development of medicinal quality. As a widely used traditional Chinese medicine, *Atractylodis Rhizoma* has a long history of medicinal application in China. However, owing to rapid overexploitation, uncontrolled reclamation and construction, and the ongoing impacts of climate change, wild *Atractylodis Rhizoma* resources have sharply declined (Jiang et al., 2023), underscoring the urgent need to identify suitable habitats. However, most existing studies on *Atractylodis Rhizoma* have focused primarily on chemical composition analysis (Chen et al., 2009) and quality evaluation (Zhuang et al., 2019). Although some studies have investigated habitat suitability zoning for *Atractylodis Rhizoma* (Ye et al., 2024), these studies remain limited and lack comprehensive and systematic analyses, particularly regarding land use types. Therefore, conducting ecological zoning studies based on land use types to predict the suitable habitats and optimal production areas of the two *Atractylodis Rhizoma* species in China under climate change holds significant practical value.

In recent years, ecological niche models have been widely used to predict the suitable distribution of species. Species distribution models (SDMs), a major class of ecological niche models, are constructed by integrating known species occurrence records with corresponding environmental variables and are widely used to characterize species ecological requirements and predict potentially suitable habitats (Sun et al., 2021; Kong et al., 2021). Owing to its advantages, including ease of implementation, robust predictive performance, and strong interpretability, the MaxEnt model has become among the most widely used SDMs and has been applied to predict suitable distribution areas and quality zoning for medicinal plants such as *Semen Armeniacae Amarum* (Ma et al., 2025), *Lycii Cortex* (Liu et al., 2025), and *Pinellia ternata* (Qiu et al., 2023). In this study, the MaxEnt model was employed, and 19 bioclimatic variables, 3 topographic variables, and 7 soil variables were selected. A quality zoning model based on key quality indicators (atractylodin content) and environmental variables was constructed. By integrating centroid migration trajectories, dynamic ellipse expansion maps, and land use types, this study systematically analyzed the key environmental factors influencing the distributions of AL and AC in China, predicted suitable habitat areas and optimal quality production regions under climate change, and provided a scientific foundation for the sustainable development of the *Atractylodis Rhizoma* industry.

## 2 Materials and methods

### 2.1 Collection of species occurrence data

**Figure 1:**
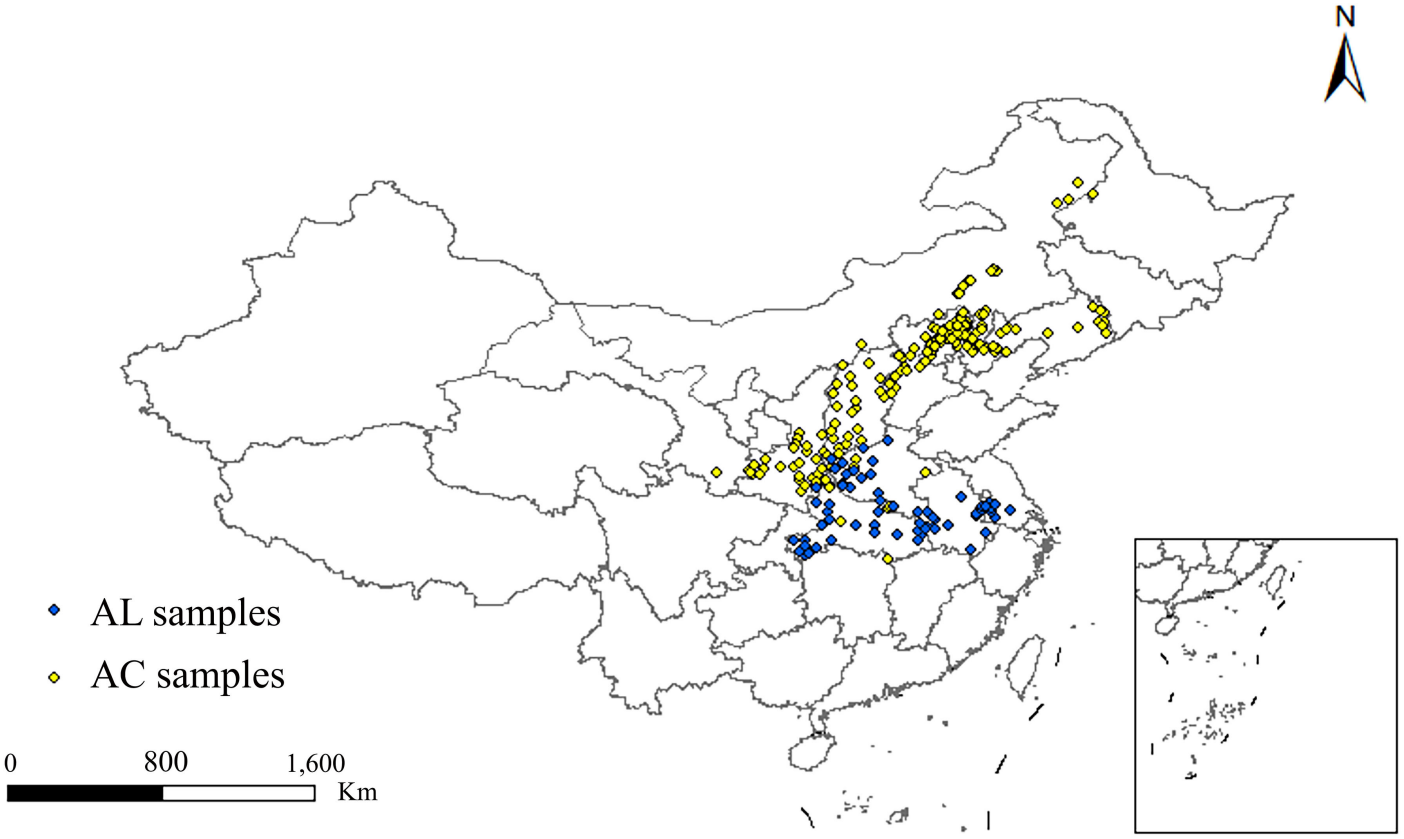
Distribution records of AL and AC in China.

Distribution records for the two *Atractylodis Rhizoma* species in this study were obtained mainly from the Chinese Virtual Herbarium (https://www.cvh.ac.cn/) and the Global Biodiversity Information Facility (https://www.gbif.org). The occurrence data for AL were mainly from Henan, Hubei, Anhui, and Jiangsu, whereas those for AC were mostly from Shaanxi, Shanxi, Hebei, Inner Mongolia, and Liaoning. To ensure the usability of the data, duplicate records and records with missing coordinates or incomplete information were removed. Owing to the large scale of this study, we referred to previous data processing methods and used the “Create Fishnet” tool in ArcGIS 10.8 to generate a 16 km × 16 km grid for spatial thinning, thereby eliminating redundant occurrence records (Boria et al., 2014; Schnase et al., 2021) and retaining only one geographical coordinate for each grid (if multiple data points were present within a grid, the data point closest to the grid center was selected). After the above screening, a total of 65 distribution records for AL and 171 distribution records for AC were retained and saved in.csv format to meet the import requirements of the MaxEnt model (**Figure 1**).

### 2.2 Environmental variables

This study used a total of 29 environmental variables (**Supplementary Table 1**), among which climate data were sourced from the WorldClim 2.1 database (http://www.worldclim.org/) (Booth et al., 2014; Fick and Hijmans, 2017), covering two periods: the present (1970–2000) and the future (2041–2060, 2061–2080). Three greenhouse gas emission scenarios from the Fifth Assessment Report of the Intergovernmental Panel on Climate Change (IPCC) were used for future climate data, namely, the RCP2.6 scenario (low concentration scenario), RCP4.5 scenario (medium concentration scenario), and RCP8.5 scenario (high concentration scenario) (Van Vuuren et al., 2011). Future climate projections were derived from the CCSM4 global climate model in the Coupled Model Intercomparison Project Phase 5 (CMIP5), which has been reported to perform well in simulating climate across China (Abdelaal M et al., 2019). Topographic data were derived from digital elevation model (DEM) data obtained from the Geospatial Data Cloud (https://www.gscloud.cn/), whereas soil data were obtained from the Geographical Information Monitoring Cloud Platform (https://www.dsac.cn/). All environmental variables were resampled to a unified spatial resolution of 1 km × 1 km, converted to the.asc format, and exported for compatibility with the maximum entropy (Maxent) model. To avoid the effects of correlations between variables that may lead to ambiguous prediction results, we performed multivariate extraction on the final collected sample data of the two *Atractylodis Rhizoma* species and conducted Pearson correlation analysis. Pairs of environmental variables with a Pearson correlation coefficient |*r*| ≥ 0.8 are considered highly correlated (Wang et al., 2023a). On the basis of the jackknife test results and the actual growth conditions of the two *Atractylodis Rhizoma* species, we excluded the factors with relatively small contributions, and ultimately, both *Atractylodis Rhizoma* species retained 19 environmental variables for MaxEnt modeling.

### 2.3 Establishment and evaluation of MaxEnt model

The species occurrence data of the two *Atractylodis Rhizoma* species and the 19 retained environmental variables were jointly input into the software, and this model was subsequently used to predict the potential suitable habitat distributions of AL and AC under current and future scenarios. We randomly selected 25% of the distribution records as the test set to evaluate the predictive performance of the model, while the remaining records were used as the training set (Hou et al., 2025). This method helps to address the overfitting problem that often occurs in maximum-entropy models, thereby increasing the predictive accuracy of the model. During MaxEnt software operation, the maximum number of iterations was set to 3000 to ensure that the model fully trained and converged, and the other parameters were presented in **Supplementary Table 2**. After multiple preruns were performed to eliminate variables with a contribution value of 0, 11 environmental variables were ultimately retained for AL, while 12 were retained for AC. After running the model 10 times, we obtained the final simulation results. In the study of predicting suitable habitat areas using the MaxEnt species distribution model, the area under the receiver operating characteristic (ROC) curve (AUC) reflects the correlation between the potential distribution probability of the species and environmental variables. Theoretically, the higher the AUC value is, the greater the prediction accuracy. Specifically, an AUC ≤ 0.5 indicates poor prediction performance; 0.5 < AUC ≤ 0.7 indicates moderate predictive accuracy; 0.7 < AUC ≤ 0.9 indicates satisfactory predictive accuracy; and an AUC > 0.9 indicates excellent predictive accuracy (Santos et al., 2019). Following the completion of the model operation and analysis, we used ArcGIS 10.8 to create a zoning map and study its suitable habitat areas. First, the average.asc data format file obtained from 10 replicate runs was converted to a raster layer; subsequently, the mapping and classification tools in ArcGIS 10.8 were utilized, with reference to the habitat suitability values of the species’ distribution points. We applied the equal-interval classification method to classify the raster layer into four suitability levels: unsuitable areas (0–0.2), poorly suitable areas (0.2–0.4), moderately suitable areas (0.4–0.6), and highly suitable areas (0.6–1) (Li et al., 2024), thus generating potential suitable habitat distribution maps for the two *Atractylodis Rhizoma* species during different periods.

### 2.4 Centroid migration and dynamic ellipse expansion analysis

We used the “mean center” and “directional distribution” tools in ArcGIS 10.8 to analyze areas of expansion, stability, and contraction, as well as centroid shifts of the two *Atractylodis Rhizoma* species under different future scenarios and time periods, and to generate maps illustrating the dynamic elliptical expansion of suitable habitats and centroid migration trends.

### 2.5 Quality regionalization

In this study, atractylodin was used as a quality indicator. On the basis of the content determination criteria outlined in the Chinese Pharmacopoeia (Commission CP, 2020), relevant data were collected from published literature (**Supplementary Table 3**). Additional screening was conducted to remove sites that were inconsistent with the known suitable distribution areas of the two species, thereby ensuring the reliability and accuracy of the data. Finally, a total of 20 AL samples and 19 AC samples with reported atractylodin contents were retained for subsequent analyses (**Supplementary Figure S1**). In this study, stepwise regression analysis was performed in SPSS 27.0 to explore the key environmental variables affecting the atractylodin content in the two *Atractylodis Rhizoma* species, with the atractylodin content as the dependent variable and the main environmental variables as the independent variables, and a relationship model was constructed using the stepwise method. We used environmental variables with a resolution of 1 km × 1 km to predict areas with high atractylodin content, which ensured the accuracy of the environmental variables at the sampling points. We used the Grid Calculator tool to construct a spatial distribution map of atractylodin content and combined it with a fuzzy overlay function and species distribution prediction maps for overlay analysis (Chen et al., 2021b), thereby generating maps of suitable habitats for AL and AC on the basis of atractylodin content under current and future scenarios.

### 2.6 Quality stable distribution areas and analysis of land use types

In this study, the common areas of quality zoning maps under different scenarios and periods were extracted through the “mask extraction method” and overlaid to construct quality stable distribution areas of the two *Atractylodis Rhizoma* species at different levels. The 2020 land use type dataset from the Resource and Environment Science Data Center of the Chinese Academy of Sciences (https://www.resdc.cn) was used to conduct an overlay analysis with the aforementioned stable distribution areas (Xu et al., 2018). After excluding land types unsuitable for the cultivation of traditional Chinese medicinal materials (arable land, urban and rural industrial and mining areas, residential land, rivers, lakes, and oceans), we constructed spatial resource distribution maps of the two *Atractylodis Rhizoma* species under different land use types and calculated the distribution areas of various types, such as dryland, grassland, forestland, shrubland, and marsh land, and sandy soil in different suitable areas to better analyze the suitable growth conditions for the two *Atractylodis Rhizoma* species.

## 3 Results

### 3.1 Model evaluation

In this study, the AUC values for AL and AC were 0.992 and 0.981, respectively (**Supplementary Figure S2**), indicating that the model can effectively predict the suitable habitat distribution of the two *Atractylodis Rhizoma* species. These results were sufficiently accurate and held considerable reference value.

### 3.2 Contribution of environmental variables

**Figure 2:**
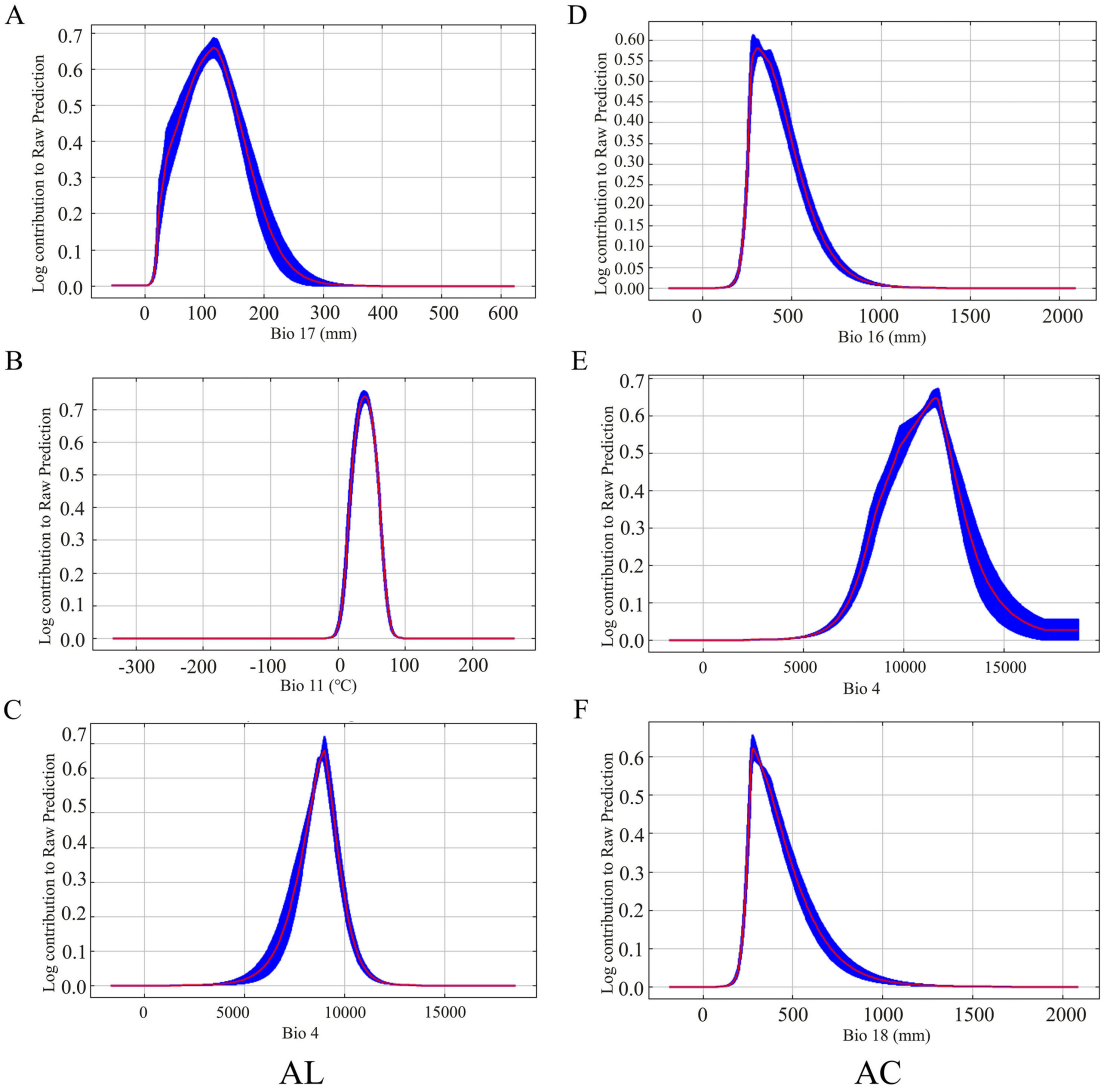
Response curves of the effects of current climatic conditions on environmental variables related to symptom distribution in AL **(A-C)** and AC **(D-F)**.

The values presented in **Table 1** represent the results of 10 replicate runs of the Maxent model for AL and AC. In terms of percent contribution, Bio17, Bio11, and Bio4 were the three environmental variables that most strongly influenced AL growth, with a cumulative percent contribution of 87.5%. In contrast, on the basis of permutation importance, Bio5, Bio11, and Bio4 were the main environmental variables governing its suitable distribution. Additionally, the results from the jackknife test (**Supplementary Figure S3A**) demonstrated that Bio11, Bio4, and Bio17 were relatively influential environmental variables. By comprehensively analyzing the response curves of key environmental variables (**Figures 2A–C**), we found that the most suitable growth conditions for AL were those for Bio17 (65 mm–150 mm), Bio11 (3°C–6°C), and Bio4 (8125–9375).

**Table 1:** Importance of major environmental variables.

Species	Variable	Percent contribution (%)	Permutation importance (%)
AL	Bio17	57.7	1.3
	Bio11	18.5	35.2
	Bio4	11.3	5.7
	slope	5.3	1.7
	Alt	2.4	0.7
	Bio5	1.4	49
	Soil G	1.1	0.2
	pH	1	0.6
	Bio18	0.9	2.8
	Bio16	0.5	2.3
	Bio3	0.1	0.5
AC	Bio16	45.4	2.2
	Bio4	19.8	17.4
	Alt	9.4	7.2
	Bio17	5.6	10
	Bio15	4.8	15.9
	Bio10	3.7	5.1
	pH	3.5	2.7
	slope	2.2	1.4
	Bio3	1.9	3.8
	Soil Texture	1.7	1.1
	Bio18	1.3	24.3
	Bio2	0.6	8.9

Analysis of the relevant AC results revealed that the three variables with the greatest percent contributions were Bio16, Bio4, and Alt, with a cumulative percentage contribution of 74.6%; the three variables with the greatest importance were Bio18, Bio4, and Bio15; and according to the jackknife test results (**Supplementary Figure S3B**), Bio3, Bio16, and Bio18 were the primary environmental variables influencing the distribution of suitable habitats for AC. Integrating these three sets of results and the response curves (**Figures 2D–F**), we found that Bio16, Bio4, and Bio18 were the key environmental variables affecting the growth of AC, with suitable values for Bio16 ranging from 260 mm–380 mm; the suitable range for Bio4 was 9000–12500, with an optimal suitable value of approximately 11500, and the suitable value for Bio18 was between 245 mm and 275 mm.

### 3.3 Suitable area prediction distribution

#### 3.3.1 Prediction of the distribution areas of the two *Atractylodis Rhizoma* species under current conditions

**Figure 3:**
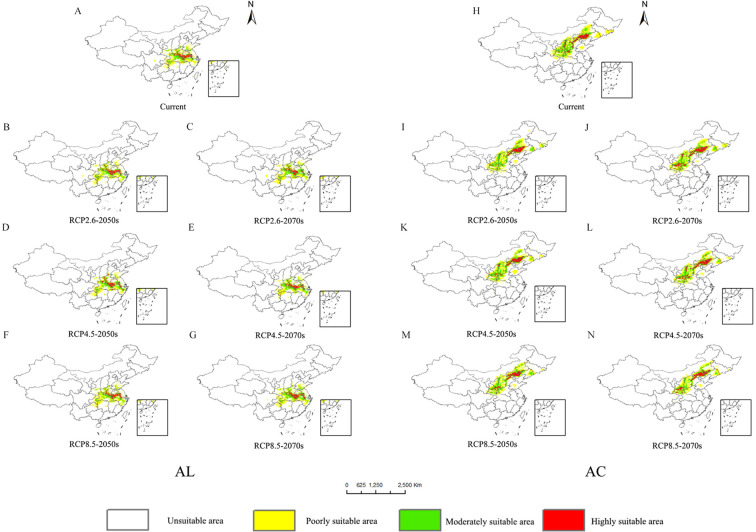
Distribution of suitable areas for AL **(A-G)** and AC **(H-N)** under current climate conditions and three climate scenarios in the future.

The suitable habitat distributions of the two *Atractylodis Rhizoma* species under current and future conditions predicted by the Maxent model are shown in **Figure 3**, and their suitable habitat areas in China were listed in **Supplementary Table 4**. The results indicated that the total area of potential suitable habitat for AL was 5.7178 × 10^5^ km^2^, accounting for 5.96% of China’s land area; its highly suitable areas were concentrated mainly in the central and southern regions of Henan, the southeastern region of Hubei, the central region of Anhui, and the southwestern region of Jiangsu (**Figure 3A**), with areas of 0.7733 × 10^5^ km^2^, accounting for 0.81% of China’s land area, while the moderately suitable and poorly suitable areas accounted for 1.89% and 3.26% of China’s land area, respectively. Under current conditions, the total area suitable for AC was 8.9691 × 10^5^ km^2^, accounting for 9.34% of China’s land area; its highly suitable area was 1.2263 × 10^5^ km^2^, accounting for 1.28% of China’s land area, and was distributed mainly in the western region of Liaoning, the central and northeastern regions of Hebei, the western region of Henan, the central and southern regions of Shanxi, the central region of Shaanxi, and the eastern region of Inner Mongolia (**Figure 3H**).

#### 3.3.2 Prediction of distribution areas for the two *Atractylodis Rhizoma* species under future conditions

On the basis of the suitable habitat distribution results for the two *Atractylodis Rhizoma* species under different future periods and emission scenarios (**Figures 3B-G, I-N**), we found that the suitable distribution area of AL mostly decreased across the different emission scenarios and future periods. In the RCP2.6-2050s and RCP2.6-2070s scenarios, the suitable area decreased by 8.50% and 1.58%, respectively; in the RCP4.5-2050s and RCP4.5-2070s scenarios, it decreased by 6.59% and 13.93%, respectively; and in the RCP8.5-2050s scenario, it decreased by 2.22%; only in the RCP8.5-2070s scenario did it slightly expand, with an increase of 2.01%; however, the overall area of highly suitable area still showed a downward trend.

Compared with the current conditions, the suitable area of AC was expected to decrease significantly in the future, with the greatest reduction occurring in the RCP8.5-2070s scenario, reaching 20.02%. Although the highly suitable area shows a downward trend, the magnitude of change is relatively small and overall stable. In the five scenarios of the RCP2.6-2050s, RCP2.6-2070s, RCP4.5-2050s, RCP4.5-2070s, and RCP8.5-2050s, the total suitable area of AC decreased by 8.17%, 8.12%, 10.52%, 15.00%, and 14.82%, respectively.

### 3.4 Centroid shifts and dynamic elliptical expansion of suitable habitat

**Figure 4:**
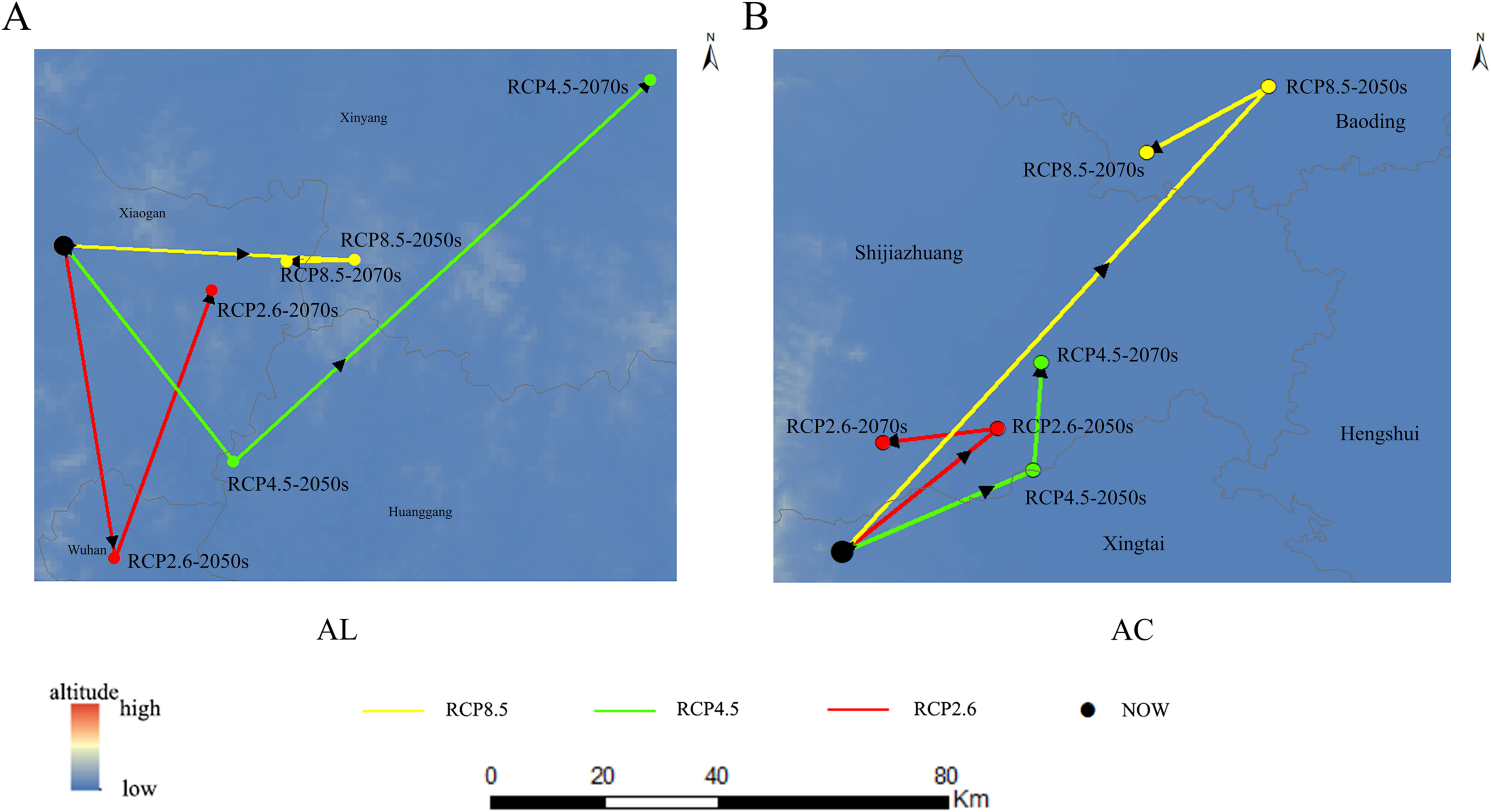
Centroid migration trajectories of AL **(A)** and AC **(B)** under different RCP scenarios and time periods.

**Figure 5:**
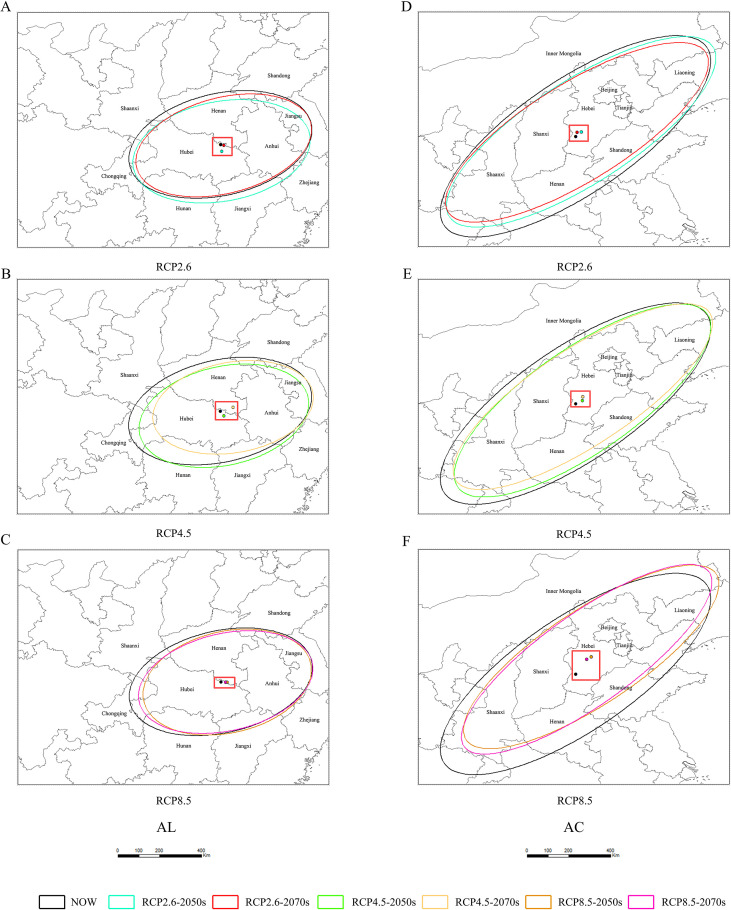
Changes in future suitability ellipses for AL **(A-C)** and AC **(D-F)** under different RCP scenarios and time periods.

The geometric centroid of the suitable habitat area was located at the center of the overall distribution and is typically used to characterize the spatial position of the population. We used spatial statistical and analytical tools to extract the centroids of suitable areas under different scenarios and periods and calculated their migration direction and distance. Additionally, using the “directional distribution” tool in ArcGIS 10.8, we obtained centroid migration paths and amplitudes, as well as dynamic ellipse expansion maps of the two *Atractylodis Rhizoma* species in their potential suitable habitat areas in China under different RCP scenarios (**Figures 4**, **5**). The results indicated that the geometric centroid of the optimal suitable area for AL was near the junction of Hubei and Henan (114.2102°E, 31.6764°N), whereas the geometric centroid of the optimal suitable area for AC was located in the southern region of Hebei (114.3006°E, 37.4299°N).

From the perspective of centroid migration trajectories, under the RCP2.6 emission scenario, AL migrated within Hubei Province. It first moved 49.24 km from Xiaogan to Wuhan and then moved back 43.72 km to Xiaogan, with the overall geometric centroid showing a tendency to migrate toward the southeast. AC first migrated 45.39 km from Xingtai and Hebei to Shijiazhuang and Hebei and then moved 23.96 km within Shijiazhuang, showing a migratory trend toward the northeast relative to the current centroid. In the RCP4.5 emission scenario, the AL and AC centroids both migrated to the northeast, reaching Xinyang, Henan (115.0342°E, 31.9078°N) and Shijiazhuang, Hebei (114.7649°E, 37.8758°N) by the 2070s, with total migration distances of 92.96 km and 72.90 km, respectively, from the current period to the 2050s and then to the 2070s. Under the RCP8.5 emission scenario, AL generally migrated in the southeast direction, first moving 38.72 km to Xinyang, Henan, and then moving 8.97 km back to Xiaogan, Hubei. Moreover, the AC still tended to migrate to the northeast in this scenario, first migrating from Xingtai, Hebei, to Baoding, Hebei, with a displacement of 149.75 km, and then migrating 30.27 km inland within Baoding.

From the perspective of dynamic ellipse expansion trends, from the current period to the 2050s and then to the 2070s, AL showed expansion trends toward the southeast, northeast, and southeast under RCP2.6, RCP4.5, and RCP8.5, respectively. The AC expanded in the northeast direction under all three emission scenarios, which was completely consistent with the predicted centroid migration trajectory trends.

### 3.5 Quality suitability analysis of AL and AC

#### 3.5.1 Relevance between environmental variables and component content

Stepwise regression analysis was used to construct two models relating the atractylodin content to environmental variables. The relationships between the atractylodin content of the two *Atractylodis Rhizoma* species and the environmental variables were as follows: Y_1_ = 22.33–0.005 × X_1_–0.21 × X_2_–0.002 × X_3_ + 0.025 × X_4_ (*p <*0.05, Y_1_ = atractylodin content of AL, X_1_ = Bio16, X_2_ = Bio3, X_3_ = Bio4, X_4_ = Bio5), Y_2_ = 1.531–0.011 × X5–0.00007111 × X_6_ (*p*<0.05, Y_2_ = atractylodin content of AC, X_5_ = Bio17, X_6_ = Bio4); the *p* values after Bonferroni correction were less than 0.01. The regression equation results indicated that Bio16, Bio3 and Bio4 were environmental variables that limited the atractylodin content in AL, whereas Bio5 increased its content within a certain range; Bio17 and Bio4 were negatively correlated with the atractylodin content in AC, indicating that both variables may suppress atractylodin accumulation.

#### 3.5.2 Quality suitability analysis of the two *Atractylodis Rhizoma* species under current conditions

**Figure 6:**
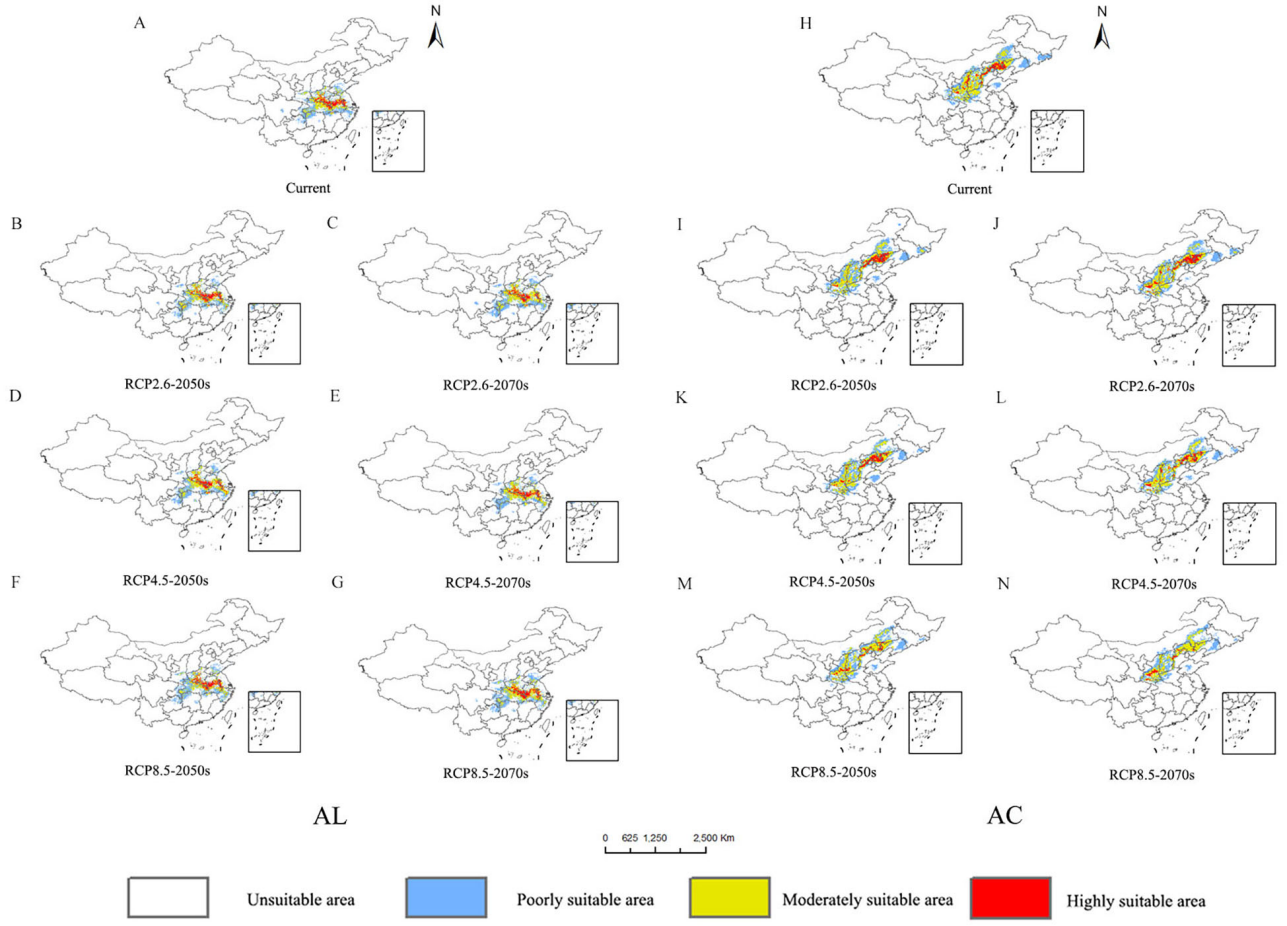
Distribution of suitable areas for AL **(A-G)** and AC **(H-N)** under current climate conditions and three future climate scenarios.

The spatial distribution and area of the atractylodin content of the two *Atractylodis Rhizoma* species under the current conditions are shown in **Figures 6A, H**; **Supplementary Table 5**. The results indicated that the total suitable area for AL was 5.7169 × 10^5^ km^2^, accounting for 5.96% of China’s land area, which was similar to the total suitable area for species distribution. The highest atractylodin content in AL was found in the central and southern regions of Henan, the northeastern region of Hubei, the central region of Anhui, and the southwestern region of Jiangsu, with areas of 0.7540 × 10^5^ km^2^, accounting for 0.79% of China’s land area. There were also some regions with relatively high atractylodin quality in Zhejiang, Hunan, Shaanxi, and Sichuan. The total suitable area for AC is 8.8679 × 10^5^ km², accounting for 9.24% of China’s land area. Compared with the current suitable area for species distribution, there was little change; however, a specific analysis revealed that the highly suitable area decreased significantly, accounting for only 0.88% of the total research area. Its highly suitable areas were concentrated mainly in central and northeastern Hebei, central and eastern Shaanxi, central and southern Shanxi, and western Liaoning and eastern Inner Mongolia.

#### 3.5.3 Quality suitability analysis of the two *Atractylodis Rhizoma* species under future conditions

From the predicted results in **Figures 6B-G, I-N**, we found that regardless of the future emission scenario and period, the change trends of the suitable area for quality and the suitable area for species distribution were essentially consistent. Overall, the total suitable area for both *Atractylodis Rhizoma* species tended to decrease relative to the current climate, except for AL under the RCP8.5 scenario in the 2070s, where slight expansion was predicted. In the future, its highly suitable areas will be concentrated mainly in the central and southern regions of Henan, the northeastern region of Hubei, the central region of Anhui, the southwestern region of Jiangsu, and the central and northwestern regions of Zhejiang. Under future conditions, the areas highly suitable for AC will be concentrated mainly in the central and southern regions of Shaanxi, parts of northern and southern Shanxi, the northeastern and central regions of Hebei, the western region of Liaoning, and the eastern region of Inner Mongolia. This area experiences a severe decline, especially under the RCP8.5 emission scenario, where the highly suitable areas in the 2050s and 2070s will be 0.4421 × 10^5^ km² and 0.3358 × 10^5^ km², respectively, accounting for only 0.46% and 0.35% of China’s land area, respectively, which is specifically reflected in the reduction in certain areas in Hebei, Shanxi, and Shaanxi.

#### 3.5.4 Quality-stable distribution areas of the two *Atractylodis Rhizoma* species

**Figure 7:**
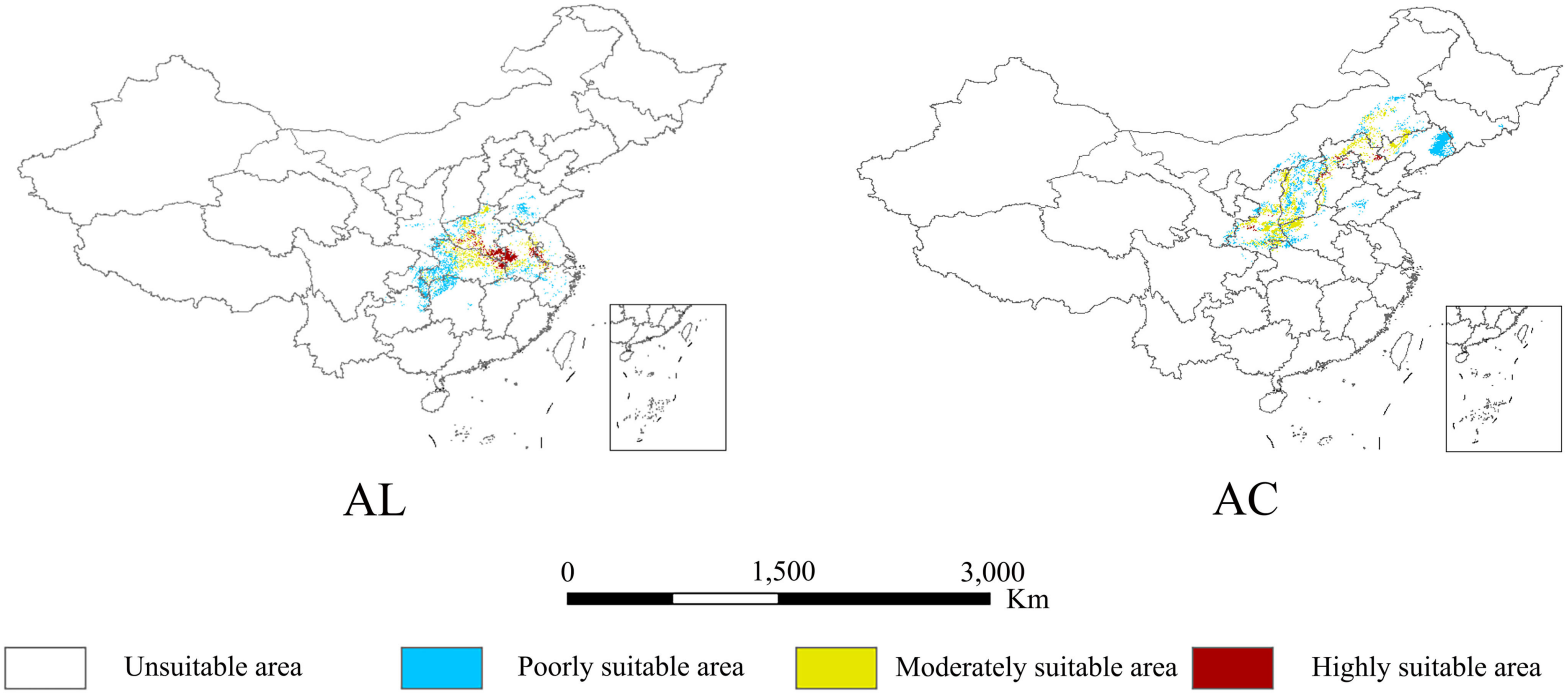
Quality-stable distribution areas of AL and AC in China.

The stable quality distribution areas of AL were primarily located in Henan, Hubei, and Anhui provinces (**Figure 7**), covering approximately 1.7402 × 10^5^ km², which represented 1.81% of China’s land area. Among these, areas classified as highly, moderately, and poorly suitable areas accounted for 0.3115 × 10^5^ km², 0.5384 × 10^5^ km², and 0.8903 × 10^5^ km², respectively. In contrast, the stable quality distribution areas of AC were primarily concentrated in Shaanxi, Shanxi, and Hebei provinces, covering approximately 1.9517 × 10^5^ km² and accounting for 2.03% of China’s land area, with highly, moderately, and poorly suitable areas of 0.0677 × 10^5^ km², 0.9301 × 10^5^ km², and 0.9539 × 10^5^ km², respectively.

### 3.6 Analysis of land use types in areas with stable distribution quality

**Figure 8:**
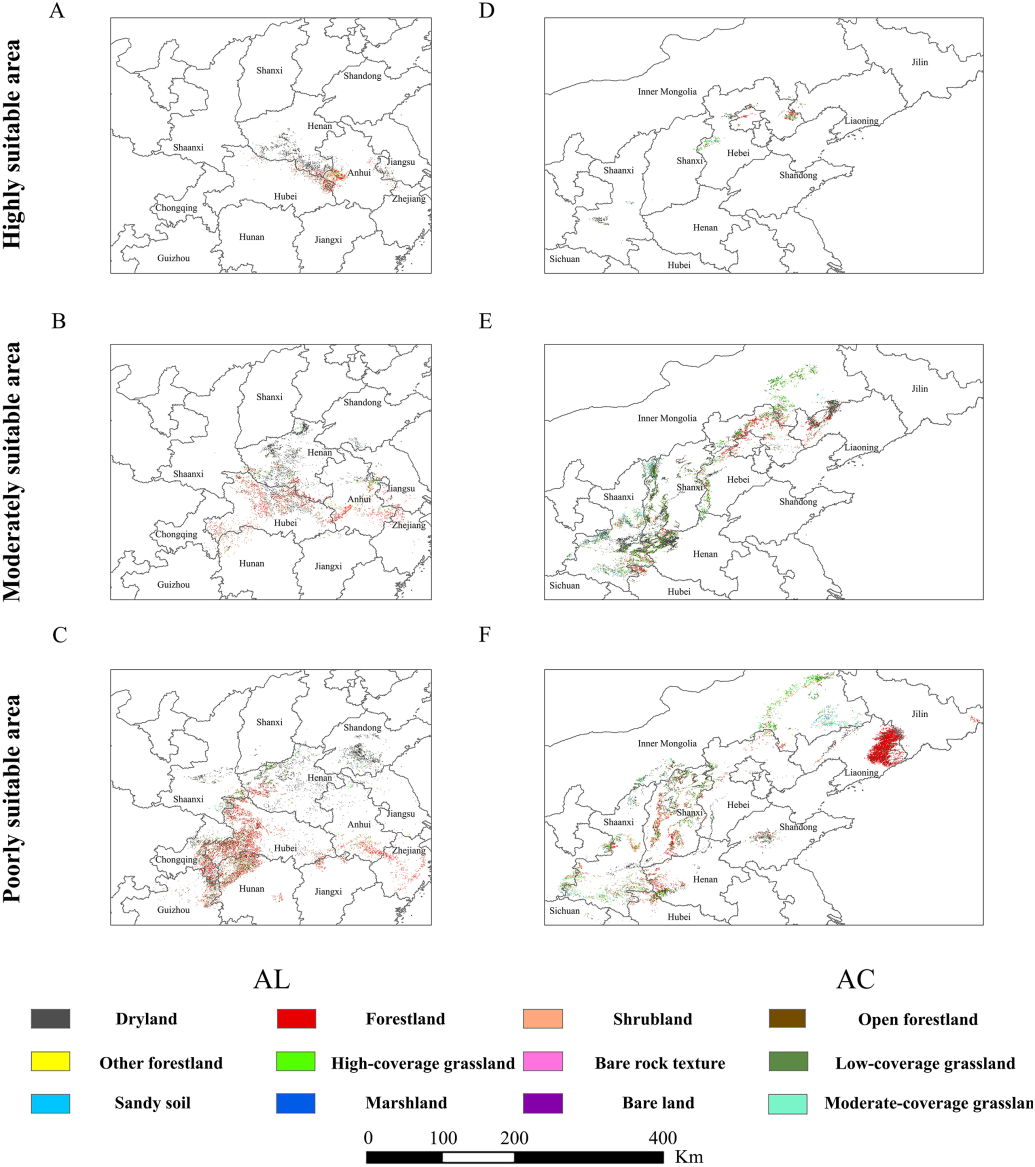
Land use types of the quality-stable distribution areas of AL **(A–C)** and AC **(D–F)**.

**Figure 9:**
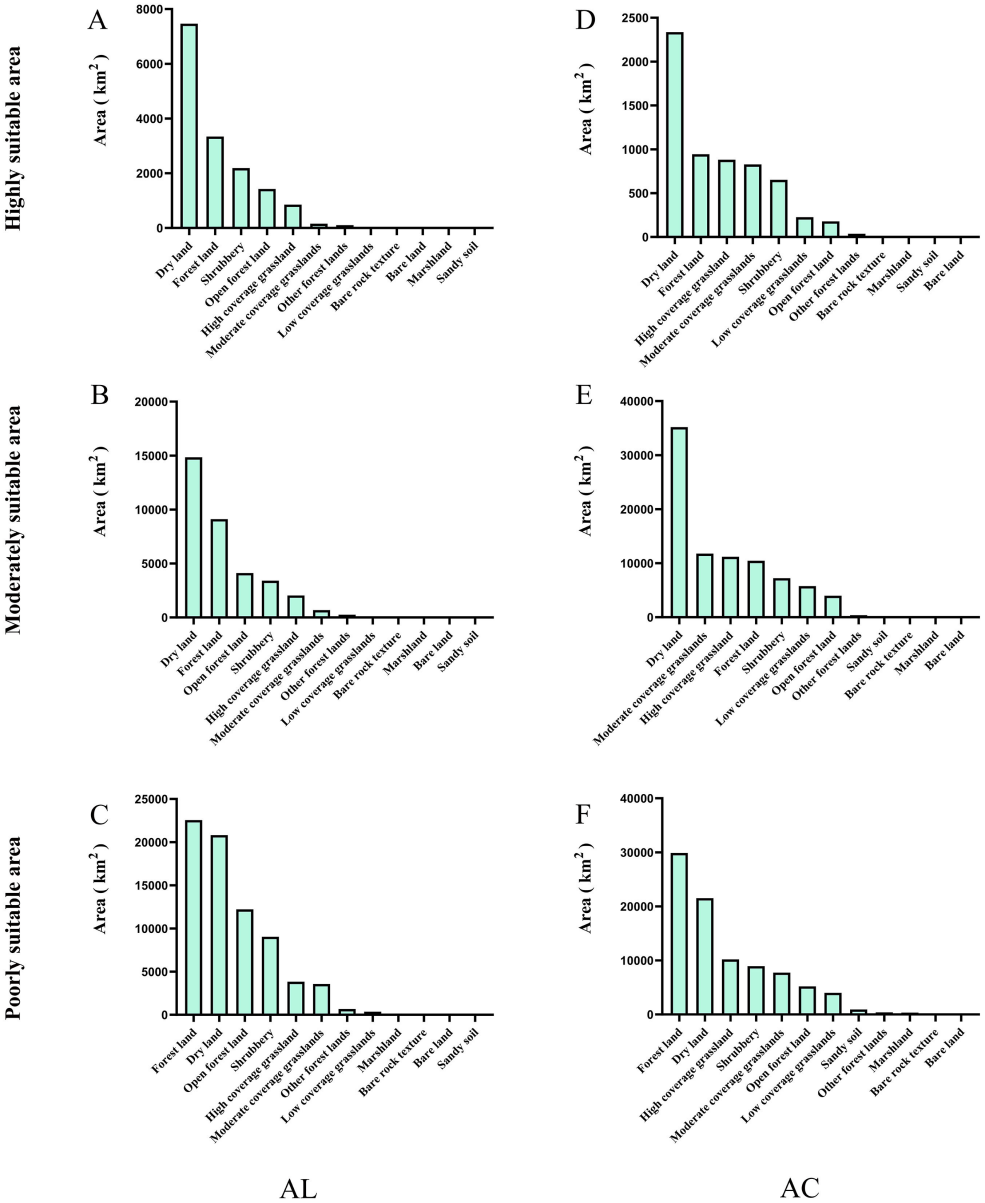
Area of land use types within the quality-stable distribution areas of AL **(A–C)** and AC **(D–F)**. Dryland (arable land without irrigation water sources and facilities, relying on natural water for crop growth), high-coverage grassland (natural grassland, improved grassland, and mowed grassland with coverage >50%), moderate-coverage grassland (natural grassland and improved grassland with coverage >20–50%), low-coverage grassland (natural grassland with coverage between 5–20%), forestland (natural and artificial forests with canopy density >30%), shrubland (low forestland and shrubland with canopy density >40% and height below 2 m), and open forestland (forestland with a canopy density of 10–30%).

This study further analyzed the main land use types of the stable distribution areas for the two *Atractylodis Rhizoma* species. The results shown in **Figures 8**, **9**; **Supplementary Table 6** indicated that the land use types of AL and AC are similar and different. Under all the climate scenarios and across all the time periods considered, dryland and forestland were consistently identified as the most suitable land use types for AL and AC. AL was also distributed in shrubland and open forestland, while AC was also suitable for planting in high-coverage grasslands and moderate-coverage grasslands. A detailed analysis revealed that dryland was the dominant land use type in both the highly and moderately suitable areas of AL and AC. In AL, drylands constituted 23.96% and 27.56% of the highly and moderately suitable areas, respectively, whereas the corresponding proportions in AC were 34.51% and 37.82%, respectively. In contrast, forestland was the dominant land use type in the poorly suitable areas of both species, accounting for 23.37% and 22.57% of the total area in AL and AC, respectively.

## 4 Discussion

The growth conditions of medicinal plants are closely linked to their geographical distribution and associated environmental variables. In the context of global warming, exploring the correlations between the distribution of medicinal plants and environmental variables, identifying key determinants of their distribution, and delineating suitable habitat areas have emerged as prominent research focuses (Bellard et al., 2012). The geographical distribution of medicinal plants is influenced by multiple factors, including climate and soil; however, climate is the dominant factor that regulates plant distribution patterns at the regional scale (Cao et al., 2019). Our results revealed that Bio17, Bio11, and Bio4 were key environmental variables affecting the distribution of AL, whereas the geographical distribution of AC depended on Bio16, Bio4, and Bio18. These findings indicate that precipitation and temperature not only are background climate variables but also influence the growth and distribution of *Atractylodis Rhizoma* by modulating the moisture–energy balance and physiological thermal tolerance thresholds within the soil–plant–atmosphere continuum. This pattern aligns with previous studies highlighting temperature and precipitation as primary variables for assessing the suitable habitats of medicinal plants using ecological niche models (Wu et al., 2024). AL is suitable for growth in warm and humid climates, which is consistent with the climatic conditions of its predicted suitable areas (Henan, Hubei, Anhui, and Jiangsu) in this study. The predicted suitable habitats for AC are concentrated mainly in Liaoning, Hebei, Henan, Shanxi, Shaanxi, and Inner Mongolia, reflecting the species’ preference for dry and cold climates (Liu et al., 2023). Notably, our predicted habitat suitability patterns were generally consistent with those reported by Wang (Wang et al., 2023b), Yu (Yu et al., 2023), and Zhang (Zhang et al., 2020), further supporting the reliability of our predictions.

With ongoing global warming, the growth and development of medicinal plants are becoming increasingly threatened, and their suitable habitats are shifting. However, the direction and magnitude of such shifts are influenced by multiple factors, including species differences and environmental complexity, leading to considerable uncertainty (Guo et al., 2020). In this study, we integrated centroid migration trajectories with dynamic ellipse expansion maps and reported that AL exhibited a southeasterly shift under both the RCP2.6 scenario and the RCP8.5 scenario; however, the magnitude of the shift is limited, and by the 2070s, AL will remain predominantly distributed in the middle and lower reaches of the Yangtze River Basin. Previous studies have indicated that most of the middle and lower Yangtze River Basin lies within the East Asian subtropical monsoon climate zone, characterized by a warm, humid climate with distinct seasons and abundant rainfall and thermal resources (Xu et al., 2024). These findings support our earlier conclusion that the AL favors warm and humid climates. Under different emission scenarios, the AC shows a northeastward migration trend, which is consistent with its preference for cold and dry environments. The migration trajectory remains largely confined to Hebei Province, and the extent of suitable habitats does not significantly change. In the future, suitable habitats for AC are projected to remain concentrated along the transition zone between semiarid and arid regions. Notably, dynamic ellipse expansion maps reveal that the distribution ellipses of the two *Atractylodis Rhizoma* species become more elongated and slightly smaller during range shifts, suggesting that suitable habitats may contract and become increasingly fragmented in the future. Although only the CCSM4 climate model was used for future projections in this study, projections generated by alternative models of species distributions can vary substantially, which may introduce uncertainty into future predictions (Araújo and New, 2007). Nevertheless, the predicted suitable habitats were generally consistent with the known distribution records of *Atractylodis Rhizoma*, supporting the reliability of the current results. Future studies incorporating multiple climate models may further improve the robustness of future projections.

The active ingredients of medicinal plants are primarily secondary metabolites, which serve as important indicators for evaluating the quality of Chinese medicinal herbs and are highly sensitive to climate, precipitation, soil, and other environmental variables (Lu et al., 2025). Contemporary global warming has altered enzyme system activity and catalytic reaction rates in medicinal plants, thereby modulating the biosynthesis of their active components (Alami et al., 2024). Our results indicated that the atractylodin content in AL was negatively correlated with Bio16, Bio3, and Bio4 but positively correlated with Bio5. These findings suggest that excessive precipitation and pronounced temperature fluctuations may inhibit the biosynthesis and accumulation of atractylodin, whereas moderately warm conditions may promote its production. This pattern may help explain the ecological adaptation of the AL to relatively warm and humid environments. In contrast, the atractylodin content in the AC was negatively correlated with Bio17 and Bio4. This relationship indicates that increased precipitation during the driest season may be unfavorable for atractylodin accumulation, whereas relatively dry conditions may promote its synthesis. This interpretation is consistent with the findings of Yu (Yu et al., 2023), who reported a negative relationship between atractylodin content in AC and precipitation. In addition, greater annual temperature variability may negatively affect atractylodin accumulation in AC. These findings indicate that precipitation and temperature are the primary environmental factors influencing atractylodin accumulation, a conclusion that is further supported by Wang et al. (Wang et al., 2023b). Previous studies have shown that moderate drought stress increases the accumulation of components such as atractylodin (Wu et al., 2026). Atractylodin is the main active compound in the volatile oil of *Atractylodis Rhizoma*, and studies have indicated that temperatures above 30°C cause seedling mortality, with high temperatures acting as key limiting factors for plant growth (Guo et al., 2005). Furthermore, temperature and its interaction with rainfall are the primary climatic factors affecting the chemical composition of *Atractylodis Rhizoma* volatile oil (Guo et al., 2007). Collectively, these conclusions align strongly with our results, further confirming the reliability of our study. In summary, global warming drives progressive temperature elevation while accelerating the hydrologic cycle and increasing precipitation in certain regions, which may alter the volatile oil composition of *Atractylodis Rhizoma*, leading indirectly to reduced atractylodin content and shrinkage of high-quality production areas.

Contemporary anthropogenic activities impact natural ecosystems with unprecedented intensity (Keck et al., 2025). The ongoing development of industrialization and urbanization has reduced the total availability of resources such as arable land and forestland, severely disrupting the land use balance for traditional Chinese herbal medicine cultivation and significantly impacting the cultivation and quality of Chinese medicinal materials. Previous studies have shown that the originally predicted suitable distribution area of the species declines significantly when coupled with land use types, with most areas having been converted to developed or arable land, thereby becoming unsuitable habitats (Rong et al., 2023). This highlights a critical limitation of traditional zoning methods that neglect human-induced land cover changes. Therefore, land use type analysis is urgently needed to accurately identify suitable habitats for medicinal plants, thereby enhancing the conservation and utilization of these resources. Barren slopes are widely distributed in hilly and mountainous regions, where the terrain is undulating and slopes are relatively steep. This makes it difficult to construct irrigation channels and other water conservancy facilities, giving these slopes the core characteristics of drylands: the absence of irrigation water sources and facilities. AL is adapted to semishad to semisunny wastelands in hilly and mountainous regions (Li et al., 2015). This growth characteristic indicates that AL is shade tolerant and intolerant of intense light, which also explains its suitability for growth on drylands as well as in shaded environments such as forestland, shrubland, and open forestland. The primary production regions of AC are concentrated in Chengde (Hebei Province) and Inner Mongolia, characterized by grassland vegetation zones, which precisely explains why AC is suitable for cultivation in areas with high to moderate grassland coverage. Notably, the land use structure in moderately and poorly suitable areas is diverse, with forestland and grassland being significantly interspersed, whereas in highly suitable areas, the available land types are relatively simple and scattered, indicating that the habitat regions may be undergoing increasing fragmentation and islandization.

In future research, we should further expand the collection range of *Atractylodis Rhizoma* samples and increase the number of sampling points to increase data representativeness and model predictive accuracy. Moreover, it is necessary to consider the potential effects of anthropogenic activities and socioeconomic development on the growth environment of medicinal plants to more comprehensively evaluate the effects of climate change on the habitat suitability and quality of *Atractylodis Rhizoma*. Furthermore, future studies should adopt an integrated modeling approach that combines the advantages of multiple niche models, which will help to more accurately predict the distribution and quality suitability of the potential habitat for *Atractylodis Rhizoma*, providing a more scientific basis for the conservation and sustainable utilization of its resources.

## 5 Conclusion

Our study evaluated the potential distribution and spatial variation of the two *Atractylodis Rhizoma* species under current and future climate scenarios and further conducted quality suitability and land use type analyses. The results indicate that under current climatic conditions, the atractylodin content is greater in both *Atractylodis Rhizoma* species growing in highly suitable areas than in those growing in other suitable regions and is influenced primarily by temperature and precipitation. Under future emission scenarios, the total area of suitable habitats and high-quality production regions for *Atractylodis Rhizoma* species are projected to decrease. These findings are based on model-based simulations rather than field-validated observations. Therefore, the results should be interpreted as indicating potential spatial patterns rather than providing definitive guidance for cultivation practices. Ecosystem conservation should be strengthened in highly suitable areas, and land use types such as dryland and forestland may be considered suitable for cultivation under appropriate conditions. Overall, this study provides a theoretical framework for understanding the potential distribution and quality variation of *Atractylodis Rhizoma* species and offers preliminary scientific support for future resource management and production planning.

## Data Availability

The original contributions presented in the study are included in the article/**Supplementary Material**. Further inquiries can be directed to the corresponding authors.
