# Supplementary material for: Integrating environmental variables and land use types to assess the habitat suitability and atractylodin content of two Atractylodis Rhizoma species under climate change
Source: Front Plant Sci. 2026 Jul 28;17:1866314. doi: 10.3389/fpls.2026.1866314 (PMC13457460; doi:10.3389/fpls.2026.1866314)
Supplement: Supplementary file 1 [file DataSheet1.docx]

Table S1 Summary of the 29 Selected Environmental Variables.

| Classification | Abbreviation | Description |
| --- | --- | --- |
| Climatic Variables | Bio1 | Annual Mean Temperature (°C) |
|  | Bio2 | Mean Diurnal Range (°C) |
|  | Bio3 | Isothermality (Bio2/Bio7) (×100) |
|  | Bio4 | Temperature Seasonality |
|  | Bio5 | Maximum Temperature of the Warmest Month (°C) |
|  | Bio6 | Minimum Temperature of the Coldest Month (°C) |
|  | Bio7 | Temperature Annual Range (°C) |
|  | Bio8 | Mean Temperature of the Wettest Quarter (°C) |
|  | Bio9 | Mean Temperature of the Driest Quarter (°C) |
|  | Bio10 | Mean Temperature of the Warmest Quarter (°C) |
|  | Bio11 | Mean Temperature of the Coldest Quarter (°C) |
|  | Bio12 | Annual Precipitation (mm) |
|  | Bio13 | Precipitation of the Wettest Period (mm) |
|  | Bio14 | Precipitation of the Driest Period (mm) |
|  | Bio15 | Precipitation Seasonality of CV |
|  | Bio16 | Precipitation of Wettest Quarter (mm) |
|  | Bio17 | Precipitation of the Driest Quarter (mm) |
|  | Bio18 | Precipitation of the Warmest Quarter (mm) |
|  | Bio19 | Precipitation of the Coldest Quarter (mm) |
| Soil Type | pH | Soil pH |
|  | Soil CEC | Cation exchange capacity of the soil (%/) |
|  | Soil sand | Soil sand content (%) |
|  | Soil clay | Soil clay content (%) |
|  | Soil G | Grade of available soil moisture content |
|  | Soil Texture | Soil Texture Classification |
|  | SOC | Soil organic carbon content (%) |
| Terrain | altitude | Alt |
|  | slope | - |
|  | aspect | - |

Table S2 The MaxEnt parameter settings.

| Species | MaxEnt parameter | Settings |
| --- | --- | --- |
| AL and AC | Regularization multiplier (RM) | 1 |
|  | Convergence threshold | 0.00001 |
|  | Feature combination (FC) | Auto features (LQPTH) |

Table S3 The data from sampling sites and the contents of Atractylodin (%)

| No. | Origin information | Atractylodin（%） | Reference |
| --- | --- | --- | --- |
| L1 | Nanhe, Hubei | 0.47 | [1] |
| L2 | Taipingfan, Anhui | 0.46 | [1] |
| L3 | Tianwang, Jiangsu | 0.64 | [1] |
| L4 | Maoshan, Jiangsu | 0.61 | [1] |
| L5 | Yangliuwan, Hubei | 0.78 | [1] |
| L6 | Taojiahe, Hubei | 0.35 | [1] |
| L7 | Hengche, Hubei | 1.22 | [1] |
| L8 | Laibang, Anhui | 0.41 | [1] |
| L9 | Luotian, Hubei | 0.40 | [2] |
| L10 | Zhufo’ an, Anhui | 0.33 | [3] |
| L11 | Sunjiayuan, Hubei | 0.36 | [3] |
| L12 | Shizhen, Hubei | 0.32 | [3] |
| L13 | Xiaohekou, Hubei | 0.38 | [3] |
| L14 | Leijiadian, Hubei | 0.46 | [3] |
| L15 | Hanpodun, Hubei | 0.47 | [3] |
| L16 | Jinpu, Hubei | 0.34 | [3] |
| L17 | Hongshan, Hubei | 0.32 | [3] |
| L18 | Jiuhua Mountain, Jiangsu | 0.32 | [4] |
| L19 | Hushan, Jiangsu | 0.48 | [4] |
| L20 | Shengli, Hubei | 0.44 | [5] |
| C1 | Mutoudeng, Hebei | 0.63 | [6] |
| C2 | Fenghuangshan, Hebei | 0.66 | [6] |
| C3 | Dihuagu, Shaanxi | 0.65 | [6] |
| C4 | Yanping, Shaanxi | 0.60 | [6] |
| C5 | Daliu, Hubei | 0.52 | [6] |
| C6 | Jiashan, Hebei | 0.64 | [6] |
| C7 | Zhangbaiwan, Hebei | 0.64 | [6] |
| C8 | Wangjiadian, Inner Mongolia | 0.47 | [6] |
| C9 | Keshiketeng Inner Mongolia | 0.53 | [6] |
| C10 | Panhe, Henan | 0.39 | [6] |
| C11 | Tai’ an, Shaanxi | 0.56 | [7] |
| C12 | Zhulinguan, Shaanxi | 0.54 | [7] |
| C13 | Huangtuwa, Beijing | 0.64 | [8] |
| C14 | Huangtuliang, Beijing | 0.61 | [8] |
| C15 | Simagou, Beijing | 0.69 | [8] |
| C16 | Pingding, Shanxi | 0.73 | [3] |
| C17 | Lanqikalun, Hebei | 0.63 | [3] |
| C18 | Fengning, Hebei | 0.68 | [3] |
| C19 | Zhalan, Inner Mongolia | 0.38 | [9] |

Note: Nos. L1-L20 were *Atractylodes lancea* (Thunb.) DC. information obtained by consulting the literature, and Nos. C1-C19 were *Atractylodes chinensis* (DC.) Koidz. information obtained by consulting the literature.

Fig. S1 Distribution point of quality prediction

Fig. S2 AUC values of *Atractylodes lancea* (Thunb.) DC. and *Atractylodes chinensis* (DC.) Koidz. in China.

Fig. S3 Jackknife results for predicting habitat distribution of *Atractylodes lancea* (Thunb.) DC. and *Atractylodes chinensis* (DC.) Koidz. under current conditions.

Table S4 Areas (×10^5^ km^2^) of suitable habitats for *Atractylodes lancea* (Thunb.) DC. and *Atractylodes chinensis* (DC.) Koidz. under future climate conditions.

| Species | Different periods | High | Moderate | Poor | Total |
| --- | --- | --- | --- | --- | --- |
| *Atractylodes lancea* (Thunb.) DC. | Current | 0.7733 | 1.8161 | 3.1284 | 5.7178 |
|  | RCP2.6-2050s | 0.7302 | 1.7062 | 2.7955 | 5.2319 |
|  | RCP2.6-2070s | 0.5813 | 1.9911 | 3.0552 | 5.6276 |
|  | RCP4.5-2050s | 0.7120 | 1.8476 | 2.7812 | 5.3408 |
|  | RCP4.5-2070s | 0.6817 | 1.7201 | 2.5194 | 4.9212 |
|  | RCP8.5-2050s | 0.7472 | 1.6360 | 3.2074 | 5.5906 |
|  | RCP8.5-2070s | 0.7563 | 1.9130 | 3.1633 | 5.8326 |
| *Atractylodes chinensis* (DC.) Koidz. | Current | 1.2263 | 2.8933 | 4.8495 | 8.9691 |
|  | RCP2.6-2050s | 1.0682 | 2.7044 | 4.4636 | 8.2362 |
|  | RCP2.6-2070s | 1.3579 | 2.7479 | 4.1347 | 8.2405 |
|  | RCP4.5-2050s | 1.1688 | 2.6390 | 4.2177 | 8.0255 |
|  | RCP4.5-2070s | 1.3602 | 2.4983 | 3.7656 | 7.6241 |
|  | RCP8.5-2050s | 1.0840 | 2.4913 | 4.0646 | 7.6399 |
|  | RCP8.5-2070s | 1.1703 | 2.1557 | 3.8475 | 7.1735 |

Table S5 Areas (×10^5^ km^2^) of suitable quality habitats for *Atractylodes lancea* (Thunb.) DC. and *Atractylodes chinensis* (DC.) Koidz. under future climate conditions.

| Species | Different periods | High | Moderate | Poor | Total |
| --- | --- | --- | --- | --- | --- |
| *Atractylodes lancea* (Thunb.) DC. | Current | 0.7540 | 1.8189 | 3.1440 | 5.7169 |
|  | RCP2.6-2050s | 0.7104 | 1.7128 | 2.8032 | 5.2264 |
|  | RCP2.6-2070s | 0.5688 | 1.9784 | 3.0780 | 5.6252 |
|  | RCP4.5-2050s | 0.6626 | 1.8692 | 2.8020 | 5.3338 |
|  | RCP4.5-2070s | 0.6667 | 1.7159 | 2.5346 | 4.9172 |
|  | RCP8.5-2050s | 0.7271 | 1.6169 | 3.2460 | 5.5900 |
|  | RCP8.5-2070s | 0.7428 | 1.8800 | 3.2028 | 5.8256 |
| *Atractylodes chinensis* (DC.) Koidz. | Current | 0.8426 | 3.2480 | 4.7773 | 8.8679 |
|  | RCP2.6-2050s | 0.7666 | 2.8339 | 4.5164 | 8.1169 |
|  | RCP2.6-2070s | 0.9880 | 2.9480 | 4.2295 | 8.1655 |
|  | RCP4.5-2050s | 0.7722 | 2.9526 | 4.1538 | 7.8786 |
|  | RCP4.5-2070s | 0.6899 | 3.0332 | 3.7931 | 7.5162 |
|  | RCP8.5-2050s | 0.4421 | 2.9634 | 4.1237 | 7.5292 |
|  | RCP8.5-2070s | 0.3358 | 2.8969 | 3.8255 | 7.0582 |

Table S6 Areas (km^2^) of land use type areas for *Atractylodes lancea* (Thunb.) DC. and *Atractylodes chinensis* (DC.).

| Species | Type | High | Moderate | Poor |
| --- | --- | --- | --- | --- |
| *Atractylodes lancea* (Thunb.) DC. | Dry land | 7463.89 | 14840.97 | 20808.33 |
|  | Forest land | 3341.67 | 9103.47 | 22544.44 |
|  | Shrubbery | 2186.81 | 3397.92 | 9029.86 |
|  | Open forest land | 1425.69 | 4113.89 | 12204.86 |
|  | Other forest lands | 99.31 | 253.47 | 661.11 |
|  | High coverage grassland | 854.17 | 2037.50 | 3819.44 |
|  | Moderate coverage grasslands | 154.86 | 679.86 | 3570.14 |
|  | Low coverage grasslands | 11.81 | 80.56 | 359.03 |
|  | Sandy soil | 0.00 | 0.00 | 2.08 |
|  | Marshland | 0.69 | 6.25 | 25.00 |
|  | Bare land | 4.17 | 2.78 | 9.03 |
|  | Bare rock texture | 4.86 | 27.08 | 23.61 |
| *Atractylodes chinensis* (DC.) Koidz. | Dry land | 2336.11 | 35177.08 | 21525.00 |
|  | Forest land | 943.75 | 10445.83 | 29874.31 |
|  | Shrubbery | 652.08 | 7227.78 | 8931.25 |
|  | Open forest land | 178.47 | 3977.08 | 5198.61 |
|  | Other forest lands | 36.11 | 385.42 | 368.75 |
|  | High coverage grassland | 880.56 | 11170.83 | 10187.50 |
|  | Moderate coverage grasslands | 827.08 | 11777.08 | 7739.58 |
|  | Low coverage grasslands | 225.69 | 5752.78 | 4000.69 |
|  | Sandy soil | 1.39 | 157.64 | 906.94 |
|  | Marshland | 2.78 | 34.72 | 327.08 |
|  | Bare land | 0.69 | 21.53 | 25.69 |
|  | Bare rock texture | 4.17 | 80.56 | 35.42 |

**Reference**

[1] Chen J. Study on Ecological Environment Herbal Quality and the Optimum Harvesting Time of the *Atractylodes lancea* (Thunb.) DC.Hubei University of Chinese Medicine. 2013.

[2] Li MY. The Basic Studies on Good Agriculture Practice of *Atractylodes lancea.* Nanjing University of Traditional Chinese Medicine. 2016.

[3] Ouyang LM. Study on the grade standard of Atractylodis Rhizoma. Shanghai University of Traditional Chinese Medicine. 2020.

[4] Xu XL, Feng X, Wang M, Ouyang Z, Xia B. A comparative study on essential oil components of wild and cultivated Atractylodes lancea. Journal of Plant Resources and Environment. 2007; (01): 28-30.

[5] Wu JX, Duan YS, Li W. Comparison on Atractylodin in the Sun-dried, Baking Bram Products of Rhizoma Areactylodis Lanceae. Hubei Agricultural Sciences. 2015; 54(08): 1974-1975+1978.

[6] Zhao YY. Chemical Pattern Identification and Antibacterial Activity of Volatile Oil of Atractylodis Rhizoma. Hebei University of Chinese Medicine. 2022.

[7] Wu XQ. Study on germplasm resources of Atractylodes lancea. Shaanxi Normal University. 2019.

[8] Meng DH, Dong SY, Song XY, Wang JF, Tian YL. Study on the Relationship Between Quality and Habitat of Wild Atractylodes chinensis (DC.) Koidz in Beijing. Tianjin Agricultural Sciences. 2023; 29(01): 84-90.

[9] Sun JK, Zhang MJ, Zhang HL, Li HL, Dong W. Determination of atractylodes in Rhizoma Atractylodis originating from differentsources by HPLC method. Journal of Xinjiang Medical University. 2021; 44(01): 102-104.
